# Dosimetric verification of micro‐MLC based intensity modulated radiation therapy

**DOI:** 10.1120/jacmp.v9i3.2832

**Published:** 2008-06-23

**Authors:** Parminder Basran, Collins Yeboah

**Affiliations:** ^1^ Department of Medical Physics Odette Cancer Centre Toronto ON; ^2^ Department of Radiation Oncology University of Toronto Toronto ON

**Keywords:** micro‐MLC, IMRT, dosimetry verification, stereotactic radiosurgery, radiotherapy

## Abstract

A methodology for the dosimetric verification of micro‐multileaf collimator (mMLC) based intensity modulated radiation therapy (IMRT) plans intended for stereotactic applications is described. The method is similar to that of conventional IMRT patient‐specific quality assurance (QA) with some notable exceptions, particularly, mechanical tests that verify the mMLC positioning with respect to the isocenter and individual leaf calibration prior to use. Relative dosimetry measurements are performed with radiographic film, a commercial film‐scanning system and a dose‐image registration program. Film dosimetry results are within ±3.0% of calculated distributions or within 2.0 mm distance to agreement. Absolute dosimetry measurements are performed with a small volume ion chamber and a commercially available stereotactic phantom. The cumulative dose from all beams is within ±2.0% of the prescribed dose. Large deviations may be observed from individual beams since the smaller IMRT fields tend to have very few high‐dose and low‐gradient regions. An independent program that examines the treatment mMLC file is used to estimate the central axis dose from each beam and provide a dose image that can be assessed alongside the intended fluence distribution prior to treatment. Tolerances for relative and absolute dosimetry of mMLC‐based IMRT treatments are tighter than what is typically reported for conventional MLC‐based IMRT. Also, the time commitment for the IMRT QA is slightly longer than of conventional MLC‐based IMRT due to QA processes that check the mechanical alignment of the mMLC device with the laser and radiation isocenter.

PACS Number: 87.53Dq, 87.53Ly, 87.53Tf

## I. INTRODUCTION

Micro multi‐leaf collimators (mMLCs) have been introduced in stereotactic deliveries as a means of increasing the dosimetric resolution of radiation dose distributions in conventional arc treatments of large, irregular tumor volumes.^(1)^ Several manufacturers now produce standalone linear accelerators with mMLCs^(2)^ or tertiary collimating mMLCs that can be attached to conventional linear accelerators.^(3)^ These technologies are particularly well suited for stereotactic radiosurgery and radiotherapy of non‐spherical tumor volumes since, theoretically, there is a reduced risk of complications when compared to conventional arc‐based therapies.^(4)^ Furthermore, these mMLC devices offer some efficiencies over conventional cone‐based stereotactic treatments since physical handling of cones is not required and the mMLC can be used in non‐stereotactic procedures.

Intensity modulated radiation therapy (IMRT) is an advanced radiotherapeutic treatment that generates highly conformal radiation dose distributions to the tumor volume while minimizing dose to peripheral normal tissues.^(5)^ More recently, these two technologies have merged resulting in more conformal dose distributions for the target and increased sparing of surrounding normal tissues.^(6)^ The adaptability of mMLCs for IMRT applications is particularly appealing since the utility of mMLCs may extend from conventional stereotactic applications to a broader range of treatment sites, such as head and neck tumors^(7)^ and prostate treatments.^(14)^

Dosimetric verification of small and complex dose distributions encountered in stereotactic treatments is challenging since the precision and accuracy requirements in patient dosimetry are more stringent than those in conventional radiation therapy. This challenge is further complicated by the irregular fluence patterns that are typical in IMRT deliveries. While there have been thorough discussions on the implementation and characterization of mMLC‐based IMRT^3^, ^6^ there is less discussion on quality assurance (QA) and dosimetric verification procedure. The tolerances of absolute dosimetry, relative dosimetry, and plan verification of these highly conformal treatments may be useful for those interested not only in implementing stereotactic delivery of IMRT, but also in more conventional IMRT treatments with mMLCs. The purpose of this report is to describe the implementation of mMLC‐based IMRT in the clinical setting and provide insight on the dosimetric verification procedures adopted in our stereotactic radiosurgery and radiotherapy practice. Since much of the methodology in the mMLC IMRT QA rivals that of conventional IMRT QA, describing the entire process would be redundant. Instead, this report will provide an overview of the procedure and highlight the key differences between conventional and mMLC‐based IMRT QA.

## II. METHODS

### A. System description

The XPLAN RT2 Stereotactic Planning system (Radionics, Tyco Health Group LP, Burlington MA) is used to plan and deliver conformal stereotactic plans with either stereotactic cones or mMLC hardware.^(3)^ This system also provides an IMRT module for generating segmented MLC fields on the mMLC.

The IMRT module is a self‐contained program that operates within the XPLAN RT2 module. The optimization algorithm is typical of that found in radiation therapy where a weighted‐quadratic function is minimized to achieve target and normal tissue dose constraints. A scaled gradient projection algorithm is adopted to estimate optimal energy fluence values.

One key advantage of the IMRT module over more conventional inverse planning algorithms is that the optimization algorithm can produce plans in several seconds, as opposed to minutes and hours, typical of conventional inverse planning algorithms. This is largely due to a simplified pencil beam dose calculation employed in the optimization. As this algorithm is different from the one used in the final calculation, differences are observed in dose distributions between the IMRT module and the clinical module. Differences in the calculated and measured doses are typically observed peripheral to the target structure in the surrounding normal tissues. Thus, to ensure adequate coverage of the target volume, a dosimetric margin of at least 2 mm, but no larger than 5 mm, is placed around the intended planning target volume. This sub‐volume provides a dosimetric margin between the high‐dose target structure and the low‐dose normal tissue.

Plan evaluation tools that are available in conventional planning are available for the IMRT plans. Thus, DVH analysis, surface dose, etc. can be performed to assess the quality of the generated IMRT plan. One unfortunate drawback of this system is that individual segments cannot be edited once exiting from the IMRT module. This makes the adjustment of individual segments impossible once the IMRT module produces the segments.

All treatment plans are delivered with the mMLC attached to a 6 MV Siemens PRIMUS linear accelerator specifically adapted for stereotactic deliveries and intensity modulated radiation. Some of the mechanical properties of the mMLC are given in Table 1.

**Table 1:** Specifications of the Micro‐MLC hardware.

*Description*	*Specification*
Number of Leaves	62
Field Size (max)	10 cmx12 cm
Leaf Width at Isocenter	4.0 mm
Leaf Thickness	7 cm
Leakage (through Leaf)	<1%
Leakage (Interleaf)	<1.2%
Maximum Leaf Speed	2.5 cm/sec
Overall Size	56×24×14 cm
Total Weight	38 kg
mMLC Control Computer	Pentium PC/Windows

### B. Relative dosimetry verification

Relative dose validation measurements are performed primarily with Kodak XV (Eastman Kodak Co., Rochester NY) sandwiched in a water phantom by comparing dose distributions delivered to the water phantom to those predicted by the software. As a part of the IMRT quality assurance, planar dose images are provided by the system where a beam delivers the intended mMLC segments to a water phantom. The dose to the phantom is calculated in a plane perpendicular to the beam's central axis at a depth of 1.5 cm, with a source to axis distance of 100 cm (or 98.5 cm to the water surface and 100 cm to the film surface). Also provided by the software are the mMLC files necessary to deliver the segments with the mMLC hardware. Monitor Unit (MU) values from each segment are linearly scaled from the treatment MU settings in order to provide a meaningful dose (and thus optical density) to the film intended for verification measurements.

To analyze the discrepancies between the measured and computed planar dose images, a commercial software was used for the IMRT comparisons initially, however, due to the fine‐precision requirements in radiosurgical procedures, the commercial system did not provide reliable dose‐registration of the measured and calculated planar dose images. Therefore, an in‐house program is used for qualitative and quantitative analysis of planar dose images. Exposed films are scanned at least 12 hours after irradiation using commercial software and after applying the appropriate optical density to dose calibration curves, the measured dose distribution is converted to a standard Tagged Image File Format (TIFF) using a VIDAR VXR‐16 scanner (VIDAR Systems, Herndon VA). After the TIFF file is created, a MATLAB‐based (The Mathworks, Natick USA) program is used for the quantitative and qualitative analysis of relative dose distributions and image registration.

The in‐house program compares the measured and computed planar dose images by 1) orienting and registering the measured and calculated dose images; 2) calculating a difference histogram and computing several statistics from the difference histogram; 3) normalizing the two images to a common point in the planar dose image; and finally, 4) displaying figures of various parameters and dose images based on the analysis. Details provided in the figures include the measured dose image, difference histogram, regions within the difference image that exceed a specific tolerance, an overlay of the measured and computed isodoses, and distance to agreement image and statistics.

The dose difference histogram^(8)^ along with the skewness of the difference histogram are computed to assess agreement between computed and measured dose images. The skewness is ascertained by calculating the percent error of the cumulative difference histogram for 5th, 18th, 82th, and 95th percentiles of the number of points (or the −2σ, −1σ, +1σ, +2σ percentiles of a Gaussian distribution). If the difference histogram is Gaussian, then the absolute value of the percent discrepancy at −2σ and −1σ will equal the absolute value of the percent discrepancy at +2σ and +1σ. The software provides the data points that correspond to the 5th, 18th, 82th, and 95th percentiles of the difference histogram and reports these values as the −2σ, −1σ, +1σ, +2σ error, respectively. Note that positive values of σ may provide negative percent discrepancies (and vice‐versa).

In high‐dose gradient regions, the distance to agreement (DTA) parameter may be more relevant since shifts in the mMLC jaw positions may result in discrepancies between measured and calculated doses. In stereotactic radiosurgery, the degree of accuracy is generally higher and thus tolerances for dose discrepancies in these regions is much lower than observed in conventional IMRT treatments. We apply the distance to agreement calculation with the knowledge that DTA criteria should be much stringent than what is typically observed in our conventional IMRT program (typically 2–3 mm). The DTA is computed in contiguous dose regions with values ranging from 20–80 % of the maximum dose.

### C. Absolute dosimetry verification

Absolute dose measurements are performed using a spherical LUCY phantom (Sandström Trade and Technology Inc., Welland, Ontario) and a PTW‐31010 (PTW‐Frieburg Germany) small‐volume ion chamber. The chamber has an inner diameter of 5.5 mm and a sensitive volume of 0.125 cm^3^. A variety of measurements were performed to obtain the necessary correction factors in order to measure the absolute dose within the acrylic phantom (see Appendix 1 for details).

A CT scan of this phantom with the ion chamber in place provides the default IMRT phantom within the XPLAN software. Once an acceptable IMRT plan is created for the patient, the user can select a quality assurance phantom for validating the dose from the intended treatment. This hybrid plan replicates the treatment fields and segments on the LUCY phantom. The isocenter of the QA IMRT plan is then repositioned to the centre of the ion chamber's active volume and the dose to the isocenter is calculated and recorded.

### D. Monitor unit verification

An important aspect of treatment planning is the verification of the treatment plan and monitor unit (MU) settings. This normally consists of checks of the gantry, table and collimator settings, prescribed dose, depths, beam weights, aperture settings, normalizations, etc. MU settings from an individual beam can be checked by calculating the dose delivered along the central axis with the given monitor unit settings and comparing the dose delivered to the prescribed dose. The depth of calculation, typically the central axis depth, the field size, and the MUs per beam is required for this check.

Since the mMLC hardware is removable, linkage of the record and verify (MOSIAQ, V1.2, IMPAC Medical Systems, Sunnyvale CA) system to the linear accelerator (the Siemens linac) is achieved by passing block codes to and from the linac and the record and verify (R/V) system. In the delivery of radiation, the DICOM RT plan that is passed to the R/V system does not include details of the mMLC leaf positions. Instead, those details are contained in a separate file residing on a Radionics mMLC computer (located in the treatment console area). This mMLC computer establishes the linkage between the R/V system, the linac, and the mMLC hardware and is responsible for providing the mMLC leaf patterns to the mMLC device. Since many commercial MU checking programs do not typically handle such MLC formats, a simple in‐house MU validation program was created using MATLAB (Mathworks, Nattick MA) that examines the MLC file used in the mMLC computer. This program uses a simple effective field size calculation for each segment, and computes the dose from each segment to the isocenter. The doses from all segments are then summed and the intended fluence map is displayed along with the calculated dose to the central axis. It should be emphasized that in this QA step, the MLC file produced by the Radionics file is examined, not the DICOM RT file passed to the R/V system since the DICOM RT file does not contain information of the mMLC leaf positions.

To demonstrate the entire IMRT QA verification procedure, two IMRT QA plans not dissimilar of those delivered on the device, are presented. The first plan (Plan 1) is a relatively large tumor volume (approximately 300 cm^3^). The intent is to deliver 3000 cGy in 5 fractions to the tumor volume. This plan consists of a 5 beam equiangular co‐planar beam arrangement where each beam delivers 11–23 segments. The second plan (Plan 2) is a small (approximately 64 cm^3^) tumor volume that was created on the LUCY phantom. This plan consisted of a 7 beam non‐coplanar beam arrangement where each beam delivers 10–12 segments. Concave normal tissues are created around the tumor volume for the dose constraints.

### E. Differences between conventional and mMLC‐based IMRT

To highlight some of the differences between conventional and mMLC‐based IMRT, we describe the steps for patient specific QA in both processes. For conventional IMRT in our clinic, step‐and‐shoot IMRT is facilitated by the Pinnacle treatment planning system (Philips Medical Systems, Cleveland, OH) on the same Siemens linear accelerator. The conventional patient‐specific IMRT QA process is essentially identical to that of the mMLC‐based IMRT QA. A hybrid plan is generated on Solid Water containing an ion chamber. Film dosimetry measurements are performed with XV film and film dosimetry is performed using similar software. The time required for both processes are compared.

## III. RESULTS

### A. Relative dosimetry verification

**Figure 1:**
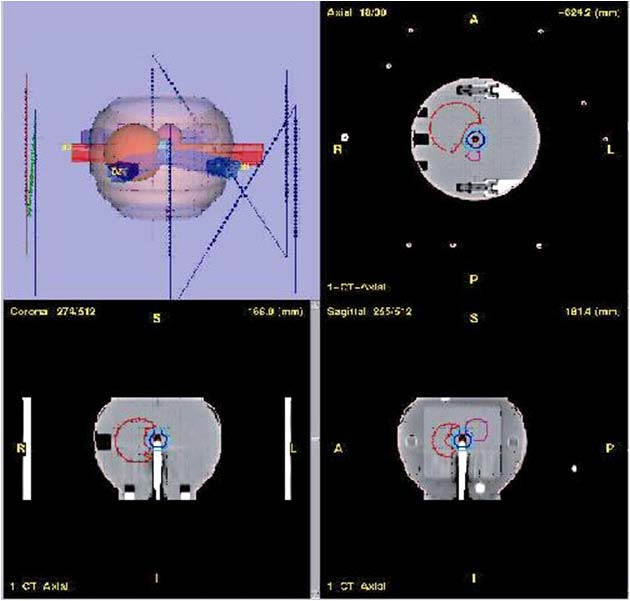
An axial (upper‐right), sagittal (lower‐right), coronal (lower‐left) and 3D view (upper‐left) of a small mMLC‐based IMRT treatment field on the LUCY phantom with the ion chamber in place.

**Figure 2:**
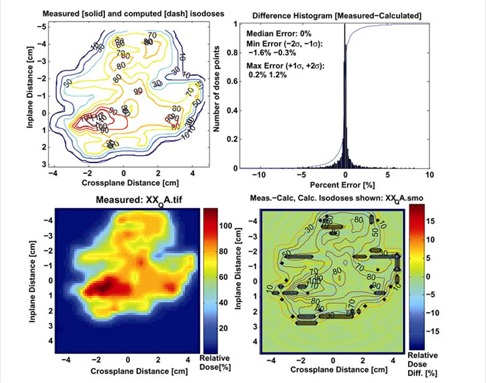
Display of the quantitative film‐dosimetry analysis. The upper right displays the measured and computed isodoses for the beam. The upper left displays the difference histogram of the measured and calculated dose distributions. The lower left displays the measured dose distribution, and the lower right displays the difference image along with regions exceeding a 3.0 % tolerance.

The percent discrepancy between measured and computed planar doses is less than 3.0% in high‐dose low‐gradient and low‐dose low‐gradient regions. Regions where the percent discrepancy exceeds 3.0% are in the high‐dose high‐gradient regions where distance to agreement is within 2 mm. Fig. 1 displays a small field IMRT plan on the LUCY phantom and Fig. 2 displays a typical printout of the film analysis from a single beam. Measured and calculated isodoses (displayed in the top left) overlap one another, suggesting excellent agreement between measured and computed dose distributions. The top‐right image displays the difference histogram along with the associated parameters that describe the spread of this distribution. The bottom right image displays a colorwash image of the difference between the measured and calculated images, where the black regions indicate those areas that exceed a 3.0% threshold.

Based on the results of approximately 15 planar dose measurements, the median, −2σ, −1σ, +1σ, +2σ errors are 0.1, −2.7, −0.6, +0.7, and 2.2 %, respectively. Distance to agreement in the high‐gradient regions is within ±1.5 mm for 95% of the data points in the high‐dose high‐gradient regions (see Tables 2and 3).

**Table 2:** Percent discrepancy for large‐sized tumor relative dose measurements.

*Beam‐Day*	*# Segments*	*Median [%]*	*Min (2σ)[%]*	*Min (1σ)[%]*	*Max (1σ)[%]*	*Max (2σ)[%]*	*DTA[mm]*
1‐1	11	−0.1	−7.5	−2.0	−0.3	1.8	1.37
2‐1	18	0.2	−0.8	−0.1	1.2	2.1	1.22
3‐1	11	−0.1	−1.8	−0.5	0.1	1.2	1.52
4‐1	23	−0.1	−2.6	−0.8	0.3	1.5	1.57
5‐1	19	−0.1	−1.6	−0.3	0.1	1.0	1.62
**Ave.**	**16.4**	**0.0**	−2.7	−0.7	**0.3**	**1.5**	**1.46**

**Table 3:** Percent discrepancy for small‐sized tumor relative dose measurements.

*Beam‐Day*	*# Segments*	*Median [%]*	*Min (2σ)[%]*	*Min (1σ)[%]*	*Max (1σ)[%]*	*Max (2σ)[%]*	*DTA[mm]*
1‐1	9	0.4	−1.1	0.0	1.4	5.8	1.47
2‐1	12	0.2	−2.2	−0.3	1.0	1.9	1.52
3‐1	14	0.1	−5.3	−1.1	0.6	1.2	1.62
4‐1	11	0.0	−7.2	−2.3	0.7	1.4	1.47
5‐1	10	0.3	−1.7	0.0	1.1	2.7	1.52
6‐1	13	0.5	−0.8	0.1	1.1	3.5	1.47
7‐1	12	0.4	−1.5	0.0	1.2	3.7	1.62
**Ave.**	**11.6**	**0.3**	−2.8	−0.5	**1.0**	**2.9**	**1.53**

### B. Absolute dosimetry verification

With the use of the LUCY phantom and applying appropriate correction factors, the LUCY phantom provides a convenient and efficient phantom for absolute dosimetry of complex IMRT plans. The ability to use the LUCY phantom for absolute dosimetry verification is particularly convenient since this phantom is routinely used for quantifying stereotactic localization errors.^(9)^

After applying appropriate dose corrections, the dose to isocenter from all beams is within 1.0% of the prescribed dose (see Tables 4and 5). However, the discrepancy between measured and calculated doses from individual beams have been measured as large as ±8.0% in some instances. Large errors are likely due to positioning of the ion chamber within straddling fluence segments of the beam.^(15)^ Clearly, placing the ion chamber in a lower‐dose gradient region would likely improve the agreement of measured and calculated doses; however, high‐dose and low‐gradient regions from stereotactic plans are more difficult to identify due to the smaller targets and mMLC leaf positions. Despite this, cumulative doses from all beams are within 1.0%.

**Table 4:** Absolute dose validation measurements for large‐sized tumor.

*Beam*	*# Segments*	*Calculated [cGy]*	*Measured [cGy]*	*Discrepancy[%]*
1	11	131.1	129.4	1.3
2	18	110.5	111.5	−0.1
3	11	110.5	114.1	3.3
4	23	123.0	126.7	3.0
5	19	129.0	127.9	0.8
**TOTAL**	**82**	**604.1**	**609.3**	**0.9**

**Table 5:** Absolute dose validation measurements for a small‐sized tumor.

*Beam*	*# Segments*	*Calculated [cGy]*	*Measured [cGy]*	*Discrepancy[%]*
1	4	117.6	118.3	0.6
2	6	121.5	124.2	−2.2
3	6	117.0	118.1	1.0
4	6	122.8	123.6	0.6
5	4	119.6	118.1	−1.3
**TOTAL**	**5.2**	**599.0**	**602.3**	**0.5**

### C. Monitor unit verification

**Figure 3:**
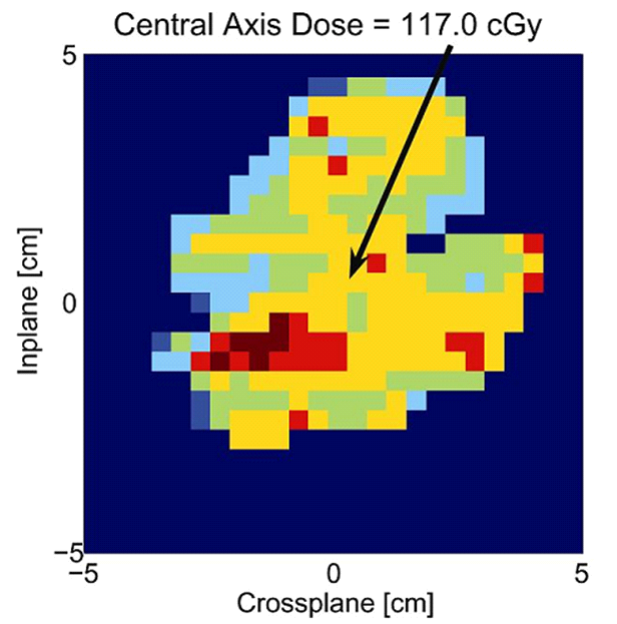
Resulting image from the MU checking program, which displays the fluence image as intended from the MLC file, along with a calculation of the central axis dose.

Independent MU validation from the treatment planning system is performed for a variety of IMRT treatment plans as well as conformal non‐IMRT treatment plans. For five patients the average dose discrepancy between calculated and measured doses from all beams is −1.7% (σ=2.0), whereas for individual beams, the dose discrepancy is −0.7% (σ=4.8%). Despite excellent agreement for the total dose to the target, the dose delivered from individual beams can be quite large. This generally occurs when there are few high‐dose low‐gradient regions in the beam. In instances where the dose to the isocenter from an individual beam is greater than 3.0%, the dose images are evaluated to ensure that there are no errors associated when interpolating the central axis dose (see Fig. 3 for an example of a dose map). Since the MU calculation is very simple, improvements in the head scatter and dose scatter could improve the algorithm significantly. Commercial vendors of MU validation software may want to consider reading the mMLC file format for plan checking purposes.

### D. Differences between conventional and mMLC‐based IMRT

The time commitment for the mMLC IMRT QA rivals that of standard IMRT practices with a few notable exceptions. The hybrid plan is very simple to generate within the planning system and the measurement time is similar to that of conventional IMRT (see Table 6 for details). Slightly more time is required to set up the LUCY phantom for the ion chamber and film measurements, as opposed to using standard Solid Water phantoms traditionally used for conventional IMRT, in order to make fine adjustments and alignments of the phantom to the radiation isocenter. Also, the mMLC must be physically installed on the linear accelerator for each use since the linac is used primarily for non‐mMLC treatments. The mMLC device itself requires a leaf calibration sequence prior to use, thereby adding a few minutes to treatment and the IMRT QA time. Also, prior to each treatment (including IMRT QA) a number of mechanical alignment tests are performed. To validate the positioning of the mMLC with respect to the isocenter and the congruence of the mMLC, projected mMLC leaf positions of a test field are compared to a physical template positioned at isocentre. The proper alignment of the mMLC device ensures not only correct beam placement, but also minimizes uncertainty of the film's registration to the expected planar dose. Finally, the laser congruence with the couch and gantry mechanical isocentre is checked prior to treatment.

**Table 6:** Timelines for patient specific IMRT QA (in hours). Note that several tests in the mMLC IMRT QA are not necessary (na) in conventional IMRT QA.

*Process*	*Conventional IMRT*	*mMLC IMRT*
MU validation and segment checks	0.5	0.5
Hybrid plan generation	1	1
Phantom set‐up	0.1	0.3
Film and ion chamber measurements	1	1
Film processing and analysis	2	2
Installing and un‐installing mMLC	na	0.5
mMLC leaf calibration	na	0.1
mMLC isocentre, light field, and field size checks	na	0.5
**TOTAL**	**4.6**	**5.9**

In total, this adds about 1.5 more hours for the patient‐specific QA when compared to the time required for our conventional IMRT patients.

## IV. DISCUSSION

Generally, tolerances for absolute dose verification measurements are within 2.0% and relative dosimetry measurements are within 3.0 % and 2.0 mm distance to agreement for mMLC‐based IMRT. This is lower than what is typically reported in the conventional IMRT literature, where relative and absolute dose verification measurements are within the 3–4% range for absolute dose measurements^(12)^ and 5% and 3 mm distance to agreement for relative dosimetry.^(13)^ In comparison, our current action levels for an acceptable ion chamber point dose validation from an IMRT hybrid measurement, film dosimetry, and relative dose distribution agreements are given in Table 7.

**Table 7:** Comparison of tolerances for conventional IMRT and mMLC IMRT.

*Metric*	*Conventional IMRT*		*mMLC IMRT*	
	*Expected Value*	*Action Level*	*Expected Value*	*Action Level*
Ion Chamber (Total)	⩽3%	>5%	<2%	>4%
Average DTA	2–4 mm	>5 mm	1–2 mm	>3 mm
Film % Error (+/−2σ)	5 %	>10%	2%	>5%

**Figure 4:**
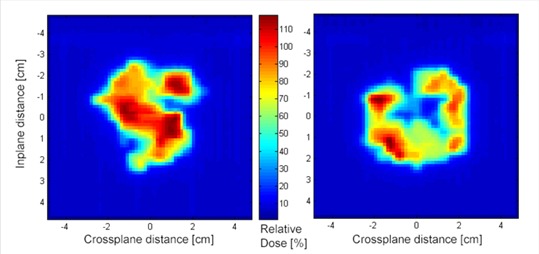
Two examples of planar dose images from a small IMRT field. Note the scale of the images.

The increase in dosimetric precision and accuracy is to be expected since the mMLC leaves are calibrated with a higher degree of precision^(3)^ and are typically a factor of two times smaller than most conventional MLCs (see Fig 4.). As a result, this increase in dosimetric precision may provide improved tissue sparing to normal tissues and improved homogeneity to the planning target volume when compared with conventional IMRT.^(14)^ However, the mMLC is limited in its application due to the smaller field sizes, and hence smaller targets, that can be treated with IMRT.

In practice, the extent of film dosimetry measurements must be moderated by realistic time constraints associated with stereotactic deliveries. Measurements of the absolute dose in the LUCY phantom and verification of the MU settings from each segment can be easily performed prior to treatment. Film dosimetry measurements, however, require time for exposure, film‐processing, and dosimetric evaluation. In our practice, film measurements are taken prior to treatment, and are evaluated the following day when a CT re‐simulation (to check for patient reproducibility) and treatment are performed. Gross errors in the intended fluence are easily detected with the MU checking program since each segment shape and beam fluence can be compared with the planning system printouts of the intended segments and fluence patterns.

## V. CONCLUSIONS

We describe a methodology for verifying the relative and absolute doses delivered from mMLC‐based IMRT treatments. This process is similar to those deployed in conventional IMRT. Our criteria for acceptable relative dose from mMLC‐based IMRT is tighter than conventional IMRT where for relative distributions the error is ±3.0% or within 2.0 mm distance to agreement. The use of auto‐registration routines of the planar doses assures a reliable and consistent comparison of the film dosimetry and calculated planar doses. Our practice is to accept a total absolute dose discrepancy of ±2.0% for the ion chamber measurements. Larger errors from individual beams are accepted if the ion chamber volume is in a high dose‐gradient region and providing that the cumulative dose from all beams remains within 2.0% of the prescribed dose. In such instances, the in‐house MU checking software is useful in identifying the positions of the high dose‐gradient regions with respect to the measurement point. The errors in the mMLC IMRT QA are smaller than that of conventional MLC‐based IMRT treatments, which suggests that more conformal dose distributions may be safely delivered with mMLC‐based IMRT.

## ACKNOWLEDGMENTS

We wish to thank Drs. Alex Lightstone and Dary1 Scora for their discussion and input and Mr. Harry Easton for his assistance with the LUCY Phantom.

## APPENDIX 1 : CALCULATION OF DOSE TO WATER IN ACRYLIC

According to the TG‐21 protocol,^(9)^ the photon spectrum and fluence at isocenter of radiologically equivalent depths in acrylic and water are identical. Thus, the dose to water, $D_{W}$, and the dose to acrylic, $D_{A}$, respectively, are related by

(A1) $$D_{A}={\left(\frac{{\bar{\mu }}_{en}}{\rho }\right)}_{W}^{A}\cdot D_{W}\cdot$$

From the TG‐51 protocol^(10)^ the dose to water is calculated from the fully corrected electrometer reading in water, $M_{W}$, using the following expression:

(A2) $$\begin{array}{rl} D_{W} & =M_{raw,W}P_{TP}P_{pol}P_{ion}N_{D,W}^{Q}\\ & =M_{W}N_{D,W}^{Q} \end{array}$$

where $N_{D,W}^{Q}$ is the absorbed dose to water calibration factor for the ion chamber. Therefore, Equation (A2) becomes

(A3) $$D_{A}={\left(\frac{{\bar{\mu }}_{en}}{\rho }\right)}_{W}^{A}M_{W}N_{D,W}^{Q}.$$

The dose to acrylic, $D_{A}$, and the fully corrected ion chamber reading in acrylic, $M_{A}$, are related by

(A4) $$\begin{array}{rl} D_{A} & =M_{A}\cdot N_{D,A}^{Q}\\ & =M_{raw,A}P_{TP}P_{pol}P_{ion}\cdot N_{D,A}^{Q}\\ & =M_{raw,A}P_{TP}N_{D,A}^{Q^{\prime }}, \end{array}$$

where $N_{D,A}^{Q}$ is the absorbed dose to acrylic per unit electrometer reading in acrylic, and

(A5) $$N_{D,A}^{Q^{\prime }}=P_{pol}P_{ion}\cdot N_{D,A}^{Q},$$

is the absorbed dose to acrylic calibration/conversion factor. Substituting Equation (A5) into the left hand side of Equation (A2) and re‐arranging for $N_{D,A}^{Q}$,

(A6) $$N_{D,A}^{Q^{'}}={\;}^{{\left(\frac{{\bar{\mu }}_{en}}{\rho }\right)}_{W}^{A}\cdot D_{W}}/M_{raw,A}\cdot P_{TP}.$$

In Equation (A6), the absorbed dose to water at a depth of 10 cm ($D_{W}$) is measured using a calibrated Farmer chamber while the ratio of mass‐energy absorption coefficient of acrylic to water is obtained from the TG‐21 report (a ratio value of 1.031 was used in this work). After having calibrated the PTW chamber in terms of dose to acrylic, the raw reading $\left(M_{\text{raw},A}\right)$ of the chamber in an acrylic phantom such as LUCY is converted to dose to acrylic ($D_{A}$) at the same point using the expression

(A7) $$D_{A}=M_{raw,A}\cdot P_{TP}N_{D,A}^{Q^{\prime }},$$

where $P_{\text{TP}}$ is evaluated using the temperature of the acrylic (i.e., LUCY) phantom. Note that while Equation (A7) is identical to Equation (A4), $D_{A}$ and $M_{\text{raw},A}$ in the former apply to an arbitrary depth and field size while a 10 cm water‐equivalent depth and a field size of 10×10 cm are used in Equation (A4) for chamber calibration. Values of $N_{D,A}^{Q\prime }$ were electrometer‐dependant and typically 29 cGy/nC for this chamber and phantom configuration.
